# Incorporation/Enrichment of 3D Bioprinted Constructs by Biomimetic Nanoparticles: Tuning Printability and Cell Behavior in Bone Models

**DOI:** 10.3390/nano13142040

**Published:** 2023-07-10

**Authors:** Tiziana Fischetti, Giorgia Borciani, Sofia Avnet, Katia Rubini, Nicola Baldini, Gabriela Graziani, Elisa Boanini

**Affiliations:** 1 IRCCS Istituto Ortopedico Rizzoli, 40136 Bologna, Italy; tiziana.fischetti@ior.it (T.F.); nicola.baldini@ior.it (N.B.); 2 Department of Biomedical and Neuromotor Sciences, University of Bologna, 40138 Bologna, Italy; giorgia.borciani@ior.it (G.B.); sofia.avnet@ior.it (S.A.); 3 Department of Chemistry “Giacomo Ciamician”, University of Bologna, 40126 Bologna, Italy; katia.rubini@unibo.it

**Keywords:** hydroxyapatite, strontium, composite hydrogel, bioink, 3D bioprinting, tissue model

## Abstract

Reproducing in vitro a model of the bone microenvironment is a current need. Preclinical in vitro screening, drug discovery, as well as pathophysiology studies may benefit from in vitro three-dimensional (3D) bone models, which permit high-throughput screening, low costs, and high reproducibility, overcoming the limitations of the conventional two-dimensional cell cultures. In order to obtain these models, 3D bioprinting offers new perspectives by allowing a combination of advanced techniques and inks. In this context, we propose the use of hydroxyapatite nanoparticles, assimilated to the mineral component of bone, as a route to tune the printability and the characteristics of the scaffold and to guide cell behavior. To this aim, both stoichiometric and Sr-substituted hydroxyapatite nanocrystals are used, so as to obtain different particle shapes and solubility. Our findings show that the nanoparticles have the desired shape and composition and that they can be embedded in the inks without loss of cell viability. Both Sr-containing and stoichiometric hydroxyapatite crystals permit enhancing the printing fidelity of the scaffolds in a particle-dependent fashion and control the swelling behavior and ion release of the scaffolds. Once Saos-2 cells are encapsulated in the scaffolds, high cell viability is detected until late time points, with a good cellular distribution throughout the material. We also show that even minor modifications in the hydroxyapatite particle characteristics result in a significantly different behavior of the scaffolds. This indicates that the use of calcium phosphate nanocrystals and structural ion-substitution is a promising approach to tune the behavior of 3D bioprinted constructs.

## 1. Introduction

Bone is a complex tissue, composed of a mineral part (biogenic hydroxyapatite), an organic part, and cells, all arranged in a highly hierarchical structure [1,2,3,4]. Understanding and reproducing the complexity of bone and of the microenvironment that surrounds the tissue, are of crucial importance for the study of physiological and pathological conditions, as well as for drug and biomaterial screening [5]. This aspect is particularly relevant in the field of orthopedic oncology, where the translation from animal models to the clinic frequently fails because of the difficulties in mimicking the complex system under investigation. For these reasons, several efforts have been devoted to the development of bone models capable of recapitulating as closely as possible the characteristics of bone, as well as a microenvironment enriched in bioactive and biomimetic stimuli to better mimic the natural microenvironment [5,6]. Such a model may be exploited in several fields, i.e., preclinical in vitro screening, drug discovery, cancer research, metabolic profiling, stem cell research, as well as pathophysiology studies, allowing the reduction in both financial and time costs [7].

In this scenario, a 3D model has to be preferred to a 2D model: 2D cultures do not fully reflect the pathophysiology of the bone microenvironment with a less reliable cell response to applied stimuli. More advanced experiments can be performed using 3D models, providing valuable insights: the cell environment can be custom-modified to mimic that of in vivo, providing more reliable cell-to-cell interactions and cell–ECM interaction and the tumor microenvironment [8,9,10].

To this aim, the use of scaffold-based techniques such as the 3D printing technology allows us to obtain 3D models in the form of hydrogel-based support to be employed as in vitro models [10,11]. The 3D bioprinting technology allows us to embed biomaterials and cells into complex 3D functional living structures, mimicking the natural ECM and recreating the natural microenvironment better than the 2D technique. For this reason, an ever increasing number of studies in the literature are being devoted to 3D bioprinting of tissue and tumor models to improve their geometry and performance [12,13,14].

In the context of bone, the inclusion of the mineral phase also has specific importance [15,16,17], on the one hand because it represents the main phase in the tissue (70 wt%), but also because it dictates the behavior of both healthy and tumor cells; hence, a mineralized model behaves differently compared to a non-mineralized one [15].

To include the inorganic phase in a 3D bioprinted construct, the use of hydroxyapatite nanoparticles is the easiest route and has been proposed in the literature [18,19,20]. Hydroxyapatite nanoparticles (nHAs) are known to improve the printability, printing fidelity, and mechanical properties of the constructs and allow us to increase the biocompatibility of the material, thus recreating a more suitable native 3D microenvironment [21,22,23,24].

However, while the majority of the studies in the literature focus on the development and application of new printing techniques and on new functionalized hydrogels, only a minority of the studies explore the use and functionalization of the inorganic phase, to tune the characteristics and behavior of the models [5]. Among these, many works use commercial HA, which prevents a fine control over the crystallinity degree, over the dimensions of the crystals, and as a consequence, over reproducibility [5], possibly increasing variability in the model behavior. Instead, the use of controlled calcium phosphate nanoparticles permits us to obtain a mineralized model having high reproducibility. At the same time, ion-substitution in calcium phosphate nanocrystals can be an easy route to tune their morphology and solubility, obtaining different in vitro performances [25]. This has important implications in 3D printing, where the shape of the particles and their solubility determine the printability and behavior of the models.

For this reason, here we focus on the use of nanoscale biomimetic particles (NPs) for the functionalization of 3D bioprinted constructs. For the first time, we exploit the different solubility and shapes of biomimetic particles having different ion-doping, to tune the printability of the inks, the characteristics of the scaffolds, and their ability to guide cell behavior. In particular, we focus on the use of stoichiometric and strontium-substituted particles. Indeed, bone apatite is a multi-substituted nanocrystalline hydroxyapatite, where multiple ions increase solubility and exert a specific biological role [3,26,27,28,29,30]. Among these ions, strontium (which substitutes for calcium) has specific importance, as it increases the number of osteoblasts and decreases the number and the activity of osteoclasts in vitro, while it reduces bone resorption and stimulates bone formation in vivo [25,31,32,33]. Bigi et al. also demonstrated how strontium can be exploited to control the shape and the dimensions of the apatitic lattice and hence of the nanocrystal [33].

Herein, for the first time, we propose the use of strontium substitution in hydroxyapatite structure to tune the characteristics of the models, by acting on particle shape and solubility. We study how to print Sr-doped and non-doped nanoparticles without negatively affecting the viability of cells embedded in the ink. In addition, we explore how the different solubility and shapes of the NPs impact on scaffold behavior, in terms of the printability, swelling, and solubility of the models, all being fundamental parameters in determining the stability and overall biological behavior of the model.

## 2. Materials and Methods

### 2.1. Synthesis of Hydroxyapatite (nHA) and Sr-Substituted Hydroxyapatite (SrHA) Nanocrystals

nHA powders were prepared by dropwise addition of 0.65 M (NH_4_)_2_HPO_4_ (50 mL) into 1.08 M Ca(NO_3_)_2_∙4 H_2_O (50 mL) solution at pH adjusted to 10 with NH_4_OH. The reaction was undertaken at 90 °C under stirring in N_2_ atmosphere; afterwards, the precipitate was left under the same conditions in contact with the mother solution for 5 h.

SrHA nanocrystals were synthesized following a similar procedure by addition of (NH_4_)_2_HPO_4_ into a 50 mL solution containing 0.864 M Ca(NO_3_)_2_∙4 H_2_O and 0.216 M Sr(NO_3_)_2_, which corresponds to a Sr/(Ca + Sr) molar ratio of 0.20.

### 2.2. Nanocrystals Characterization

Ca and Sr contents in the solid products were determined by means of an Agilent 4210 Molecular Plasma-Atomic Emission Spectrometer (MP-AES) (Agilent Technologies, Santa Clara, CA, USA). Powders were previously dissolved in 0.1 M HCl.

X-ray diffraction analysis was carried out by means of a PANalytical X’Pert PRO powder diffractometer equipped with an X’Celerator detector (Malvern Panalytical Ltd., Malvern, UK). CuKa radiation was used (40 mA, 40 kV). The 2θ range was investigated from 10 to 60 2θ degrees with a step size of 0.1° and time/step of 100 s.

A Philips CM100 transmission electron microscope (Philips, Amsterdam, NL, USA), operating at 80 kV, was used for morphological observations. Apatitic powders were suspended in ethanol after sonication and then were transferred onto holey carbon foils supported on conventional copper microgrids. Starting from the TEM images, particle size was measured by averaging at least 90 measures taken from >10 separate and non-overlapping particles. Length, width, and aspect ratio were measured.

### 2.3. Preparation of the Ink and Set-Up of the Printing Parameters

To investigate the effect of the ceramic phase alone and its capability to tune the properties of any polymeric ink, we selected a commercial ink, Alginate-RGD ink (A-RGD, SKU: IK2000110301) (CELLINK, Göteborg, Sweden). An alginate-based ink was selected, also incorporating an RGD motif, as it is among the most employed natural polymers in 3D bioprinting applications, thanks to its low cost, biocompatibility, and overall properties (solubility, porosity, degradability, and viscosity), which can be tuned by adjusting the concentrations [7,11].

Different nHA and SrHA particle concentrations were considered and added to A-RGD ink to find the optimal concentration suitable for the 3D printing process: 0.5%, 1%, 2% *w*/*v*. The following abbreviations are used throughout the manuscript (Table 1):

The nHA and SrHA particles were added to the alginate ink and the solution was mixed under stirring for 30 min at room temperature and further sonicated for 15 min to allow optimal homogenization. The extrusion-based 3D printing process was conducted using an extrusion bioprinter (BioX, Cellink, Sweden). Samples were square structures of 5 × 5 × 1 mm^3^, composed of two layers printed with a 22 G (410 µm) blunt tip nozzle at 25 °C. The 3D printing parameters were optimized and are reported in Table 2. Immediately after printing, the structures were cross-linked with 100 mM CaCl_2_ for 2 min at room temperature. Printing parameters were selected based on preliminary data of optimal printability (data non-reported).

### 2.4. Measurements of 3D Printing Accuracy—Alginate w/wo Particle Functionalization

The printing accuracy of the 3D alginate structure with and without particle characterization was investigated to detect any differences between pure alginate and alginate functionalized by nHA/SrHA particles. Briefly, a grid (10 × 10 mm) with a 5% infill density (0–90° pattern) was printed for each condition. To calculate printing accuracy, *n* = 3 grids were printed, and macroscopic images were acquired. For each printed grid, *n* = 3 measurements were performed on the inner fibers (*n* = 2) by ImageJ software (version 1.54d) and compared to the nozzle dimension (Ø = 410 µm) to obtain accuracy %, calculated by Equation (1):

(1) $$Accuracy\;\left[\%\right]=\left[1-\left|\frac{D_{theoretical}-D_{measured}}{D_{theoretical}}\right|\right]\times 100$$

The accuracy was considered acceptable for values ≥ 65% [34,35].

### 2.5. Viability Pre-Screening

Prior to performing the 3D bioprinting experiment, a pre-screening test with bulk material (50 µL of ink) manually mixed with 5 × 10^3^ Saos-2 cells was performed to evaluate the effect of nHA and SrHA particles on cell viability at different time points (1, 3, 7, and 14 days). Tests were carried out with Alamar Blue, as described below.

### 2.6. Physical Characterization: Swelling Test Study and Ion Release Profile

#### 2.6.1. Swelling Test Study

For the swelling test, cylinders were obtained and weighted (*W*_0_), then placed in 12-multiwell plates (*n* = 5 per condition, conditions Alg, Alg1nHA and Alg1SrHA), immersed in 3 mL of 1 M HEPES buffer (7.5 pH) and stored at 37 °C. For this study, only Alg and Alg1nHA and Alg1SrHA were considered to analyze the effect of the higher NPs concentrations compared to the pure alginate. At defined time points (10 min, 30 min, 1 h, 3 h, 18 h, 24 h, 72 h, 168 h, 336 h), samples were removed from the HEPES buffer, gently swabbed with tissue, and weighted (*W_t_*). The percentage weight variation ∆*W* [%] was evaluated by comparing the sample weight at the different time points (*W_t_*) to the initial weight (*W*_0_) using the following Equation (2):

(2) $$∆W\;[\%]=\frac{W_{t}-W_{0}}{W_{0}}*100$$

#### 2.6.2. Metal Ions Release

Release of Ca and Sr was measured in 1 M HEPES solution (pH = 7.5). Each sample was immersed in 2 mL of HEPES solution and the supernatant liquids were removed from the wells at increasing times up to 14 days and stored at T = −20 °C until analysis. Metal content at each time point was analyzed by means of an Agilent 4210 MP-AES (Agilent Technologies). The strontium line at 460.733 nm and calcium line at 616.217 nm were used. The calibration lines were made with 4 calibration standards (Sr: 0.5, 2, 5, 10 mg/L; Ca: 0.5, 5, 10, 20 mg/L), prepared by dilution of 1000 mg/L strontium or calcium standard solutions in diluted HNO_3_. Results from this analysis represent the mean value of three different determinations.

### 2.7. Cell Expansion and 3D Bioprinting Experiment

Saos-2 human osteoblast-like cells were purchased from ATCC. Cells were maintained in Iscove′s Modified Dulbecco′s Medium (IMDM, Gibco, Thermo Fisher Scientific, Milan, Italy) with 1% penicillin–streptomycin (penicillin 10.000 units/mL, and streptomycin 10 mg/mL—Euroclone, Milan, Italy) and 10% fetal bovine serum (FBS, Sigma-Aldrich, St. Louis, MO, USA) in a humidified incubator at 37 °C in a 5% CO_2_ humidified atmosphere and the medium changed every 3 days. For passaging trypsin/EDTA (Trypsin 0.05%–EDTA 0.02% in PBS, Euroclone, Milan, Italy) was used.

For the bioprinting process, 1 × 10^7^ Saos-2 cells homogeneously suspended in 100 µL of complete medium were mixed with 1 mL of the ink and place into a syringe. By means of an adapter, the bioink was transferred into a cartridge equipped with the 22 G (410 µm) blunt tip nozzle and the 3D bioprinting process was performed. The bioprinting process was performed following the parameters reported in Table 2.

### 2.8. Alamar Blue Assay

To assess the presence of metabolically active Saos-2 cells, the one-step Alamar Blue assay (Invitrogen, Carlsbad, CA, USA) was performed on the bulk materials (5 × 10^3^ Saos-2/sample) and bioprinted samples, according to the manufacturer’s instructions. Briefly, the culture medium was removed, replaced with the Alamar Blue solution prepared as 10% *v*/*v* in fresh cell culture medium, and incubated at 37 °C, 95% humidity for 4 h. Then, the fluorescence of Alamar Blue solution was quantified using a microplate reader (Infinite F200 PRO, TECAN, Mannedorf, Switzerland) at 535 nm excitation and 590 nm emission wavelengths. The hybrid material without cells was analyzed and considered as background.

### 2.9. Live Dead Assay

To visualize and quantify the presence of viable and dead cells, the Live Dead assay (LIVE/DEAD™ Cell Imaging Kit (488/570), R37601, Invitrogen, Thermo Fisher Scientific, Milan, Italy) was performed according to the manufacturer’s instructions. Briefly, after the removal of the culture medium, the samples were washed with PBS and the staining solution was incubated in an orbital shaker for 20 min at 37 °C, protected from light. After rinsing with PBS, the samples were examined under the optical fluorescent microscope (NikonTI-E, Nikon Corporation, Amsterdam, The Netherlands) and representative images were acquired at 20× magnification using the NIS-Element image software BR4.00.00 (version 4.40, Nikon). 

Five representative fields were acquired for each sample and manually analyzed by counting viable (green) and dead (red) cells, and the percentage of viable cells as the ratio of total live/dead cells was calculated for each sample at 1, 3, and 7 days. The experiments were performed twice and on triplicate samples.

At the same time, dual-photon confocal microscopy observation was performed at early time points to estimate cell viability and observe cell distribution in the full-thickness samples. Images were acquired by using an A1R MP dual-photon confocal microscope (GaAsP) and NIS-Element AR software (Nikon) v5.40.01. We used an optical path mode and a custom channel series mode, with a first dichroic mirror IR-DM, a first filter cube 492 SP, a second filter cube 525/50, a third filter cube 575/50, and an objective 25× immersion with a numerical aperture 1.1, refraction index 1.333, resonant scanning, z-step 2 µm. For the acquisition parameter, we used laser wavelength 1000 and power 22.5, for all the channels, PMT HV 138, PMT Offset −5 for R-CH2, PMT HV 134, PMT Offset −10 for R-CH3, line average 4, pinhole size 255.4 µm, scan speed 7.5, zoom 1.0. To automatically count the % of viability (live/dead ratio), we used the Bio Analysis tool of the NIS-Element AR software (version 5.40.01) and quantified the average value obtained for each section of a total z-section of 500 µm.

### 2.10. Statistical Analysis

Data are expressed as mean ± standard error of three replicates, with *p* ≤ 0.05 considered as statistically significant. Statistical comparisons between the experimental groups and between the different time-endpoints were made by a nonparametric Mann–Whitney test for unpaired data, using the StatView 5.01 for Windows software (version 10 Pro, SAS Institute Inc., Cary, NC, USA) for viability assays. Repeated measure ANOVA was used for weight variation tests.

## 3. Results

### 3.1. Characterization of the Nanoparticles

**Figure 1:**
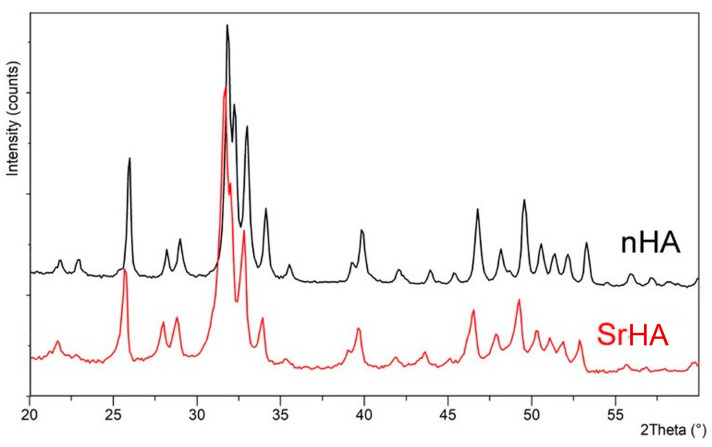
Powder X-ray diffraction patterns of nHA and SrHA nanocrystals. Sr substitution in the HA structure results in shifted XRD peaks.

The powder X-ray diffraction patterns of nHA and SrHA displayed the reflections characteristic of hydroxyapatite as a single crystalline phase (PDF 9–432), as shown in Figure 1. Sr substitution for Ca in the HA structure provoked a shift of the XRD peaks toward lower angles because of an increase in the cell parameters that was caused by the larger dimensions of the substituting cation. This shift was in agreement with the amount of Sr incorporated into the nanocrystals, which accounted for 18 at%. Our previous studies showed that Sr content up to about 10 at% in the composition of SrHA had a beneficial effect on bone cells, significantly stimulating osteoblast activity and differentiation. Moreover, at low concentration, Sr affected osteoclast proliferation, which was progressively reduced with increasing Sr content [36,37]. Similar Sr amounts were able to improve the biological performance of HA in vivo, representing a promising strategy, especially in osteoporosis patients with high risks of spinal fusion failure [38]. Herein the higher Sr content (18 at%) was chosen, taking into account that SrHA nanoparticles were embedded into the polymeric ink, which was expected to cause inhibition of the solubility of the apatitic crystals.

**Figure 2:**
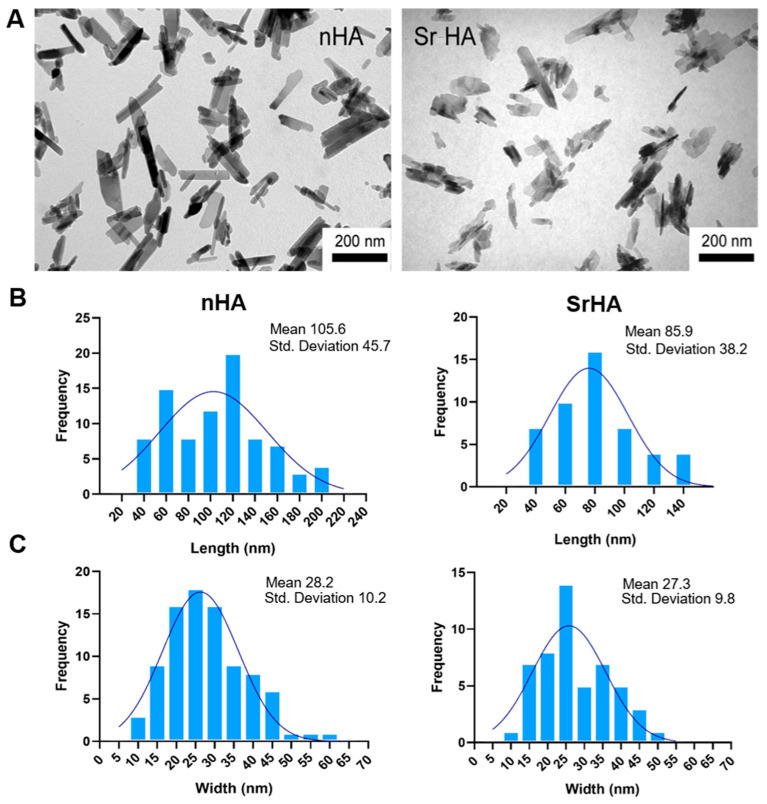
(**A**) TEM images of nHA and SrHA nanocrystals. Scale bar (200 nm) is the same in the two images, for direct comparison. (**B**) Length distribution of nHA and SrHA, (**C**) width distribution of nHA and SrHA.

Moreover, the dimensions of SrHA nanocrystals were smaller and their shape less defined than those of the HA, as can be observed in the TEM images reported in Figure 2A.

To perform a quantitative comparison between nHA and SrHA nanoparticle dimensions, the distribution of length, width, and the aspect ratio were measured (Figure 2B,C). nHA nanocrystals had a length (40–200 nm) and width (10–60 nm) higher than SrHA (40–140 nm length and 10–50 nm width). Thus, the length/width aspect ratios of nHA and SrHA were 1.5–7 for nHA and 1.5–3.5 for SrHA, with this difference being statistically significant (*p* < 0.01).

### 3.2. Alginate Functionalization with Hydroxyapatite Nanocrystals

nHA and SrHA particles at different concentrations were combined with alginate materials and tested for 3D printing process feasibility once we evaluated the absence of the cytotoxic effect of the sterilization method on cell viability. A 2% *w*/*v* concentration resulted in nozzle-clogging for both nHA and SrHA, causing excessive particle aggregation and subsequent inhomogeneity and disaggregation from the hydrogel. The data of the preliminary assessment of ink biocompatibility and cell viability in the presence of the particles are in the Supplementary Materials, Figure S1. Based on the scarce printability for SrHA, nHA and SrHA 2 *w*/*v*% were excluded from the study. Instead, 0.5% and 1% *w*/*v* nHA and SrHA in the gel resulted in suitable printability and were selected for the 3D bioprinting experiments.

**Figure 3:**
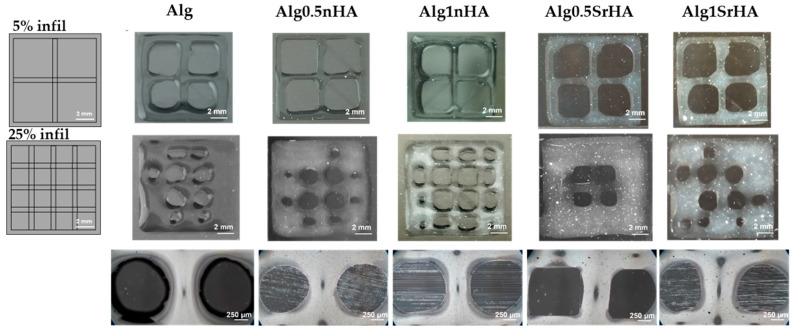
Alg, Alg0.5nHA, Alg1nHA, Alg0.5SrHA, and Alg1SrHA, at different infill densities. (**Top**) CAD design of grid with 5% infill density and results obtained for each condition. (**Middle**) Grids with 25% infill density and results obtained for each condition. Scale bar: 2 mm. (**Bottom**) Pore appearance obtained for each condition of the 3D bioprinted structures. Scale bar: 250 µm.

In Figure 3 (top) we show the 3D printed structures obtained with a 5% infill density, whereas the ones in Figure 3 (middle) were obtained with a 25% infill density. The appearance of the pores in the Alginate-RGD matrix was observed after the 3D printing process, and is shown in Figure 1, bottom. From a qualitative evaluation, it can be observed that open pores were obtained for all the conditions with the 5% infill density, while some pores collapsed and merging was noticed when increasing the infill to 25%. Fiber dimensions obtained with the 5% infill and printing accuracy [%] were calculated for each condition and are reported in Table 3. In pure alginate (Alg), the printing accuracy [%] was close to the minimal acceptable range, with fibers starting to merge at 25% infill density. In Alg0.5nHA, we observed the same behavior as pure alginate, although the fibers with 5% infill density were closer to nozzle dimension, thus leading to increased printing accuracy. In Alg1nHA fiber dimensions were close to the nozzle dimension in the 5% infill, and we observed marginal merging fibers effect at the 25% infill density and only in the border areas. Thus, the addition of 1% nHA resulted in higher coherence to the theoretical design. Regarding 0.5% and 1% SrHA, particle aggregation was observed on the images acquired by optical microscopy (Figure 3, bottom). Fiber dimensions in the SrHA printed structures were similar to the alginate ones, although closer to the nozzle dimension and with a higher printing accuracy. Thus, the addition of particles had a positive effect on the 3D printing accuracy and shape retention. Moreover, printability and printing accuracy were affected by the particle shapes, with the needle-like nHA showing a better effect.

### 3.3. Swelling Test

**Figure 4:**
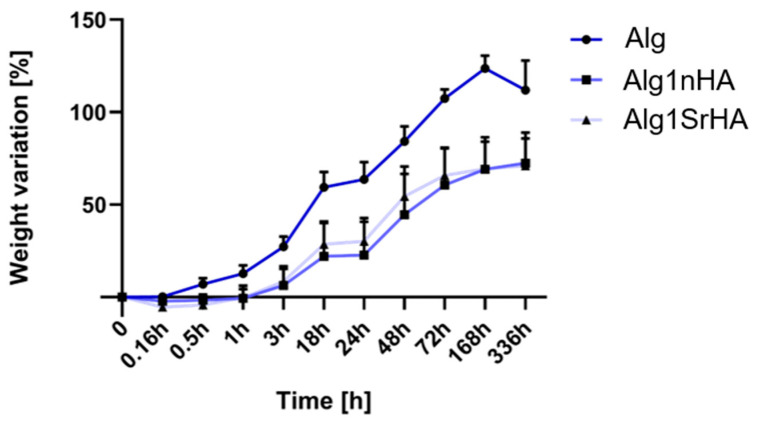
Weight variation for Alg, Alg1nHA and Alg1SrHA conditions at different time points.

The samples immersed in HEPES for 14 days showed a progressive increase in volume and weight over time (Figure 4). Alg samples started swelling after 10 min immersion, and weight variation (124 ± 7%) continued to increase up to 7 days. After this time and up to 14 days, alginate underwent a weight loss of 10%. This weight loss was imputable to Alg dissolution and, in detail, to the exchange reactions occurring between the Ca^2+^ cross-linker ions and the cations (i.e., Na^+^ and K^+^) present in the culture medium. However, Alg dissolution was moderate and did not affect the 3D structure stability. In Alg1nHA samples, swelling started after 1 h and continued up to 7 days (22% increase up to 1 day and 70 ± 17% up to 7 days), with the maximum weight variation up to 3 days. After 7 days, the weight stabilized until day 14. Similarly, in Alg1SrHA samples the weight started to increase after 1 h and continued up to 7 days, with lower weight variation after day 3 (30 ± 12% increase up to 1 days and 70 ± 15% up to 7 days), and weight stabilization from day 7 on. Comparing the different conditions, Alg samples had a more pronounced and continuous weight variation compared to both Alg1nHA and SrHA samples, with this difference significant at all the considered time points (*p* < 0.01). Alg1nHA and SrHA followed a similar trend, as differences in weight variations were not statistically significant for any of the considered time points.

### 3.4. Metal Ions Release

**Figure 5:**
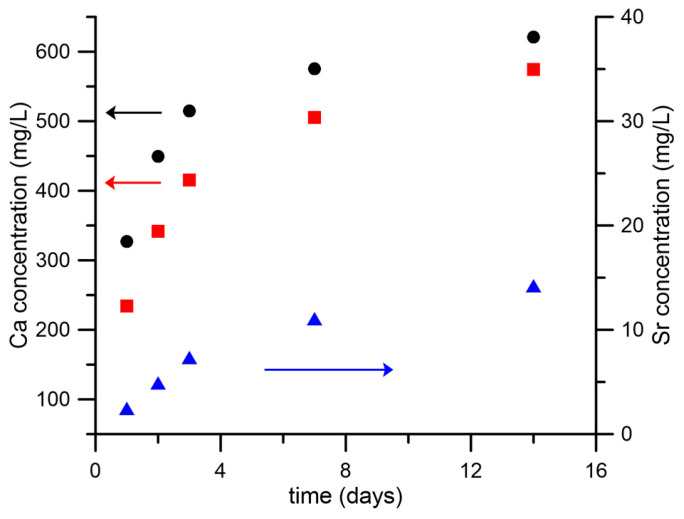
Calcium and strontium cumulative release from scaffolds containing nHA or SrHA powders as a function of soaking time in HEPES solution. Calcium release is reported from nHA (black ●) and SrHA (red ◼); strontium release from SrHA (blue ▲).

The rate and amount of ion release can affect cell response. Therefore, we investigated Ca and Sr release from scaffolds containing nHA and SrHA in the buffer solution. The cumulative ion release is reported in Figure 5 as a function of time up to 14 days. Ca release from nHA was about 330 mg/L after 1 day, and it increased up to 620 mg/L at 14 days. These quantities were always higher than the amounts of Ca released from SrHA after the same periods of time, which was not surprising because of the stoichiometric composition of the crystals. The amount of Sr released from SrHA was significant. Nonetheless, the sum of Sr and Ca moles released from SrHA was always lower than the extent of Ca released from HA, which apparently was in contrast with the higher solubility of Sr-substituted HA [36]. Actually, Ca release from the alginate scaffold (not containing particles and used as reference material) was about 170 mg/L after 1 day, which meant that part of the Ca used for scaffold cross-linking was partially released in the solution as well.

In general, ion release from the NPs was low, as expected. Indeed, NPs were added to the inks at a maximum concentration of 1 *w*/*v*% and embedded in the alginate matrix, which reduced the specific surface exposed to the medium. In addition, ions were not immediately available, but needed to diffuse through the alginate in order to be released in the medium. Finally, both stoichiometric and Sr-doped hydroxyapatite had low solubility. Further studies are in progress to investigate if the use of more soluble phosphates (such as brushite and monetite), leading to higher ion release, can be beneficial.

### 3.5. Cell Viability Evaluation

#### Saos-2 Cell Viability and Distribution in the 3D Bioprinted Samples

**Figure 6:**
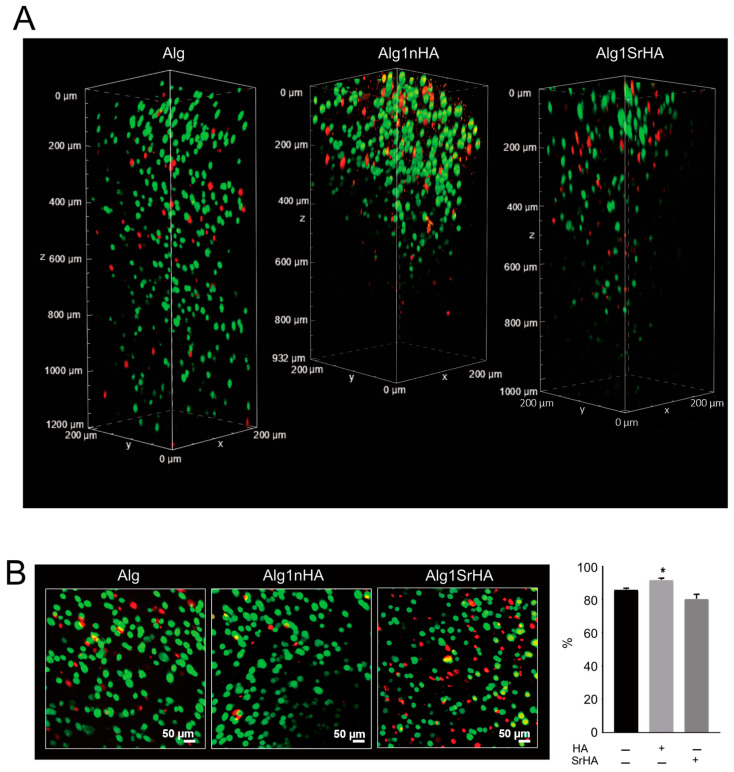
Saos-2 cell viability on pure Alg and Alg1nHA and Alg1SrHA performed by Live/Dead staining and acquired by dual-photon confocal microscopy at 24 h. (**A**) Volume render; (**B**) intensity projection (left panel, scale bar 50 µm) and % of cell viability automatically quantified (right panel, medium ± SE, * *p* < 0.05, *n* = 3 for Alg and Alg1nHA, and *n* = 4 for Alg1SrHA).

Samples were observed under confocal microscopy to evaluate cell distribution along the entire thickness of the samples following printing (Figure 6). The different levels of transparency of the scaffolds, resulting from the presence of the NPs, affected the observation of the inner areas of the scaffolds. However, at 24 h, confocal microscopy analyses of samples revealed that cell distribution was homogeneous within the entire sample thickness, even at the higher NP concentration.

Overall, cell viability was high for all conditions and was not negatively affected by the presence of the NPs. Notably, an increase in cell viability was observed for Alg1nHA samples. Cells distribution was also homogeneous, even in the deeper areas of the samples, indicating that the addition of particles, even at the highest concentration, did not negatively affect the distribution of cells or cause excessive stress during printing.

**Figure 7:**
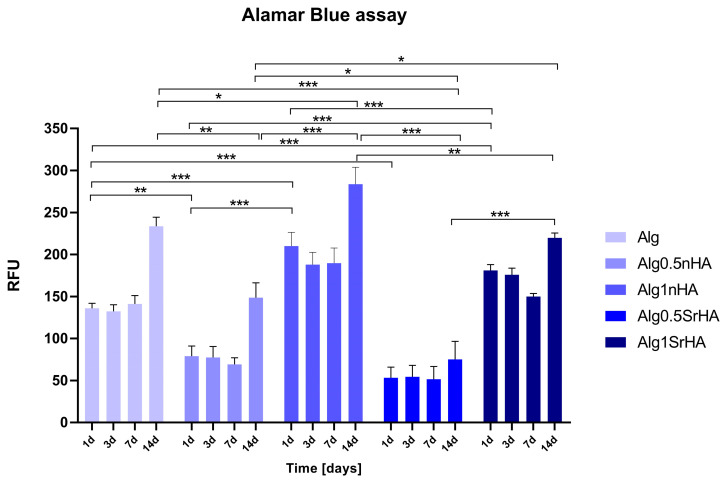
Metabolically active Saos-2 cells in 3D printed samples at all the considered time points (1, 3, 7, and 14 days) (* *p*-value ≤ 0.05, ** *p* ≤ 0.01, *** *p* ≤ 0.001).

Bioprinted sample were maintained in culture until 14 days, and an Alamar Blue assay was performed to assess the metabolically active Saos-2 cells. Data collected are reported in Figure 7. Cell viability was maintained in all samples up to 14 days, with a positive trend of growth overtime. Alg1nHA and Alg1SrHA had higher values of cell viability in comparison to Alg0.5nHA and Alg0.5SrHA and to alginate in the absence of the particles (Alg). This behavior was not noticed for the non-printed inks, indicating a positive effect of the nanoparticles (at the higher concentration) on the printing behavior and on cell viability at longer time points. Interestingly, scaffolds functionalized with nHA showed a better behavior compared to the SrHA counterpart.

**Figure 8:**
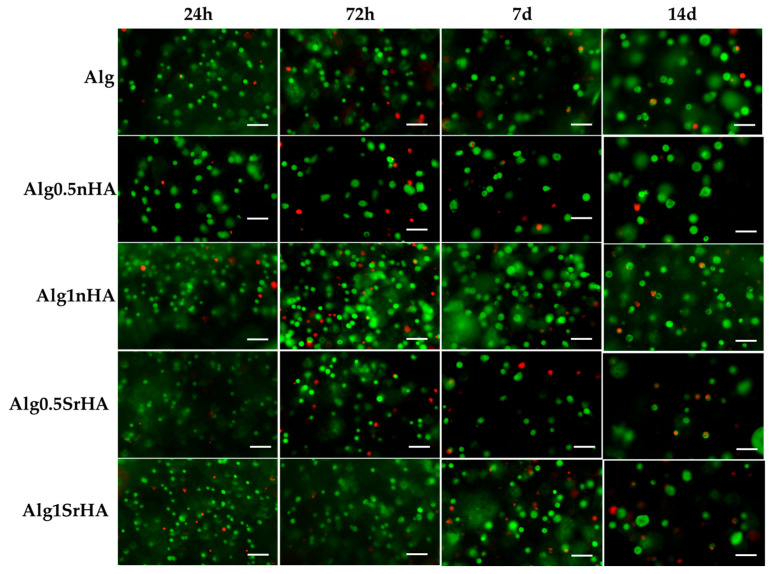
Representative images acquired by optical microscopy of Saos-2 cells embedded in the inks and stained with Live Dead assay at all the considered time points (1, 3, 7, and 14 days). Scale bar 100 µm.

**Figure 9:**
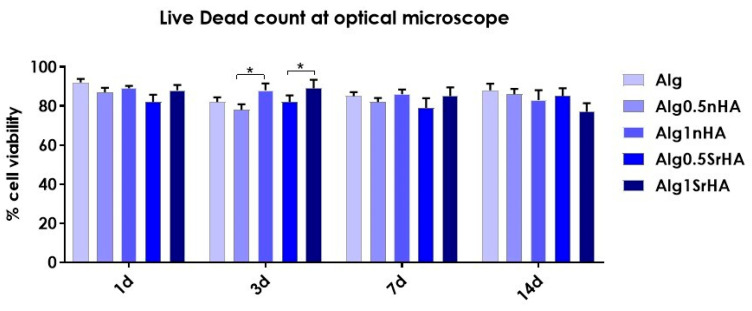
Saos-2 cell viability (quantified by Live/Dead staining, acquisition by optical microscopy) on 3D bioprinted samples composed of pure alginate and alginate + nHA and SrHA particles at different concentrations, over 14 days (* *p*-value ≤ 0.05).

The 3D printed samples with Saos-2 cells were also evaluated by Live Dead assay to visualize viable and dead cells embedded in the inks, and representative images of samples are reported in Figure 8. The count of live and dead cells and the conversion in percentage of cell viability were as reported in Figure 9. In all samples, a good cell viability was found, with live/dead ratio values higher than 75% of viable cells for all conditions and time points.

High viability at early time points (>90% for all conditions) indicated that, even in the presence of the particles, the cells did not undergo excessive shear stress, confirming the results of the metabolic activity. At longer time points, we observed a constant trend in viability, with an increase at 3 days for Alg1SrHA and for Alg1nHA, which was the one showing the best performance also in terms of metabolic activity. However, at 14 days, Alg1SrHA samples showed a slight reduction in cell viability, although not statistically significant. Nevertheless, since the viability was above 75% for all inks, they could all be considered suitable for the proposed application. Although viability was high for all inks, in the case of alginate alone, samples at 14 days tended to fracture and become difficult to collect by a spatula, hence showing the worst behavior among the samples.

Cell images in Figure 7 and counts in Figure 8 suggested that the overall number of cells decreased at 7 and 14 days, thus contradicting data obtained by Alamar Blue. However, quantification of the number of cells was hampered by the fact that cells were distributed within the full thickness of the sample and the optical microscope could acquire only one focal plane. In additon, at 14 days, we observed the formation of spheroid-like structures. This was clearly detectable by comparing the dimensions of single cells at 24 h with those of clusters at 14 days. We inferred that the cells did proliferate but tended to organize in clusters instead of randomly distributing in the ink, because of the physical and mechanical constraints imposed by the alginate, which hampered their migration, or because of increased proliferation around NP clusters. This aspect is the object of further investigations, but it confirmed, rather than contradicting, data obtained by Alamar Blue, which gave an indirect number of the cells present in the ink by evaluating their overall metabolic activity. In fact, Alamar Blue data showed that the metabolic activity at 14 days increased, suggesting that the number of cells was maintained quite homogenously over time but their metabolic activity increased.

## 4. Discussion

In this study, commercial RGD-enriched alginate was combined with hydroxyapatite nanoparticles with and without Sr substitution for the development of an osteomimetic ink to obtain a 3D biomimetic bone tumor model.

To improve the biomimicry of the Alginate-RGD and to enhance the biomimicry and behavior of the final ink, the addition of custom-made nanoparticles of stoichiometric and Sr-substituted hydroxyapatite was considered. The particles differed for ion-doping, which resulted in a different morphology and solubility (Figure 1 and Figure 2). Indeed, HA constituted plate-shaped crystals, with mean dimensions up to about 200 × 40 nm, whereas SrHA nanocrystals displayed more perturbed shapes, ill-defined edges, and slightly smaller mean dimensions.

All NP-doped inks, at all concentrations of both n-HA and SrHA, were biocompatible, with the absence of cytotoxic effects.

Regardless of the particle type, a moderate number of particles can be added to the ink to avoid dishomogeneity and needle clogging. In our case, we found the optimal addition to be 1% *w*/*v*. Our results were similar to what was found in the literature regarding HA for 3D bioprinting applications, which found the printable range to be between 1% and 5%, with significant needle clogging above this concentration [39]. Instead, we were able to incorporate a significantly higher amount of SrHA, since the literature data indicated scarce printability above 0.1–0.75 wt% [39,40].

Above this level, in our experiments, an excess of particles also interfered with the long-term viability of cells inside the ink, possibly owing to excessive density interfering with nutrient exchange. For this reason, this condition was discarded.

At 1% *w*/*v* and below, instead, the addition of the inorganic phase allowed us to control the printability of the ink and its physico-chemical characteristics while maintaining a good biocompatibility. Indeed, addition of stoichiometric apatite resulted in increased printing fidelity (expecially at the high values of the infill) and reduced swelling. From the comparison between nHA and SrHA, nHA particles had higher printing fidelity compared to the alginate and SrHA particles. This could be related to their different shapes: the needle-like morphology characterizing nHA particles could ease the extrusion of the hydrogel throughout the nozzle and positively influence the post printing shape maintenance. Conversely, the spherical morphology characterizing SrHA particles could result in particle agglomeration, hampering the gel flowing out of the nozzle and leading to higher inhomogeneity in the deposited material.

Regarding swelling, the effect of the NPs in opposing swelling could be noticed after 0.16 h (vs. the Alg group, where a 15% weight variation was observed up to 1 h) and over 14 days. This effect depended on the fact that NPs distributed within the free polymer chains, thus occupying free spaces for liquid absorption. As a consequence, although HA is hydrophilic, the effect of the NPs filling polymer chains prevailed and swelling inhibition was observed during the first hour of immersion. Then, the trend between NP-loaded and -unloaded samples was the same, with initial swelling observed for all samples until 7 days, when dissolution of the ink prevailed, causing a decrease in the overall sample weight. No particle-dependent effects were noticed, since the curves of the two NP-loaded inks overlapped.

Comparing the two particles, we observed a different ion release, which was higher for stoichiometric HA, contrary to what was expected based on particle solubility. However, in our case, the measured amount of Ca in the solution was partially ascribed to the CaCl_2_ used for sample cross-linking. The higher amount of residual cross-linker suggested that nHA samples had a lower degree of reticulation, so this aspect will need further characterization. However, in our case, nHA 1% was the sample showing the best biological behavior, especially in terms of metabolic activity, indicating that cells were not released from the scaffold, as would occur in the case of insufficient cross-linking.

A biological evaluation was then performed to assess the effects of NP-HA and Sr-NP-HA in the 3D printed constructs on Saos-2 cell viability. We found that both particles could be printed without affecting cell viability. Indeed, high metabolic activity was obtained over time by the Alamar Blue assay for NP-HA and Sr-NP-HA at both 0.5% and 1%, and an increasing trend in cell proliferation was observed from early time points until 14 days of culture, especially for NP-HA. In general, the behavior was better for the inks with 1 wt% of nanoparticles, where the effect of the bioactivity of apatite prevailed over the shear stress caused by the presence of particles during printing. In fact, while viability remained high for all inks, a reduction in metabolic activity was found for samples with 0.5% NPs compared to 1 wt% NPs, regardless of the particle type, which indicated that cells experienced some stress during printing. In addition, the behavior was better for stoichiometric apatite, which showed a cell viability higher than Sr-NP-HA, indicating an important effect of particle geometry and solubility, even for moderate differences in these parameters. Indeed, the needle-like shape of the HA particles appeared more suitable for printing. These data were also confirmed by the Live Dead assay: the direct quantification of cell viability by counting viable cells and dead ones showed values over 90% for all samples at 24 h, with confocal microscopy showing a slightly higher viability for samples with 1% nHA. In addition, a homogeneous cell distribution was found, with no areas of low viability being observed, not even in the central part of the samples.

After 14 days of culture, all samples had a viability > 75%, with alginate samples showing higher variability and lower maintenance of shape and integrity, which indicated a worse and faster dissolution behavior. The reported results suggested that the presence of both nHA and SrHA particles can enhance cell clustering, compared to alginate alone. These preliminary results suggested that the addition and modulation of an inorganic phase in the inks can be a cheap and easy route to dictate the behavior of tumor cells in the models and their capability of forming clusters and spheroids, so further studies are in progress to better study this phenomenon.

In light of using the scaffolds as 3D bone tumor models, we showed that the incorporation of nanoparticles did not only permit biomimicry, but also obtained a better behavior in terms of printing fidelity and swelling. Printing of NPs at a concentration up to 1 wt% was permitted without any damage to cell viability upon printing, up to late (14 days) time points. Instead, it helped to obtain greater stability of the scaffold. Significant differences were observed when ion-doping was taken into account, hence changing the morphology and solubility of the particles, opening the possibility of printing particles with a wide range of ion-doping for different applications.

In our study, the goal was to investigate the possible application in bone tumor models, but the results we obtained for the use of biomimetic NPs to guide scaffold behavior and obtain mineralized tissue models also appear promising for other applications in healthy tissues, such as bone or cartilage, or for non-oncological pathologies. However, further studies will be needed to extend this model to different applications, to ensure that biocompatibility is also preserved for other cell lines. However, since the cytotoxic effect toward Saos-2 was negligible, we expect a high biocompatibility also for non-tumor bone cells (i.e., MSCs, osteoblasts).

## 5. Conclusions

In this study, we proposed a new route to customize the physical properties and the in vitro behavior of 3D bioprinted tumor models by adding biomimetic calcium phosphate nanoparticles having different ion-doping. We showed that, independent of the type of particles, a 1% *w*/*v* concentration can be added to the ink while maintaining optimal extrudability and biocompatibility.

At this concentration and below, the addition of NPs to the ink increased printing fidelity, in a particle type-dependent fashion, and reduced swelling, regardless of particle size and shape. At the same time, no reductions were observed in cell viability after printing or at longer time points (14 d) for any of the conditions and in any area of the samples, indicating that no detrimental effects derived from particle addition. Instead, the addition of non-soluble and needle-like stoichiometric apatite permitted us to increase biomimicry of the models, favoring cell viability at short and longer time points.

These results thus demonstrate that the addition of tuned NPs is a suitable and simple way to customize inks for 3D bioprinting. The characteristics of the model are strongly affected by small modifications in the particle characteristics; hence, they can be finely modulated. These 3D in vitro models of bone tumor with high viability at early and late time points can be exploited in several field of research.

## Figures and Tables

**Table 1:** Abbreviations used for the different hydrogel combinations throughout the text.

Full Name of the Hydrogel	Abbreviation
Alginate-RGD	Alg
Alginate-RGD + 0.5% *w*/*v* nHA	Alg0.5nHA
Alginate-RGD + 1% *w*/*v* nHA	Alg1nHA
Alginate-RGD + 2% *w*/*v* nHA	Alg2nHA
Alginate-RGD + 0.5% *w*/*v* SrHA	Alg0.5SrHA
Alginate-RGD + 1% *w*/*v* SrHA	Alg1SrHA
Alginate-RGD + 2% *w*/*v* SrHA	Alg2SrHA

**Table 2:** Printing parameters optimized for each condition.

Hydrogel	Pressure [kPa]	Speed [mm/s]
Alg0.5nHA	28–35	6
Alg1nHA	30–35	6
Alg2nHA	30–40	6
Alg0.5SrHA	23–25	6
Alg1SrHA	23–25	6
Alg2SrHA	40–42	6

**Table 3:** Fiber dimensions measured for the different conditions: Alg, Alg0.5nHA, Alg1nHa, Alg0.5SrHA, Alg1SrHA.

Condition	Fiber Dimension	Accuracy [%]
Alg	556 ± 40 µm	66
Alg0.5nHA	532 ± 47 µm	71
Alg1nHA	501 ± 58 µm	79
Alg0.5SrHA	541 ± 43 µm	69
Alg1SrHA	524 ± 55 µm	73

## Data Availability

The data presented in this study are available on request from the corresponding author.

## Supplementary Materials

The following supporting information can be downloaded at: https://www.mdpi.com/article/10.3390/nano13142040/s1, Figure S1: Metabolically active SAOS-2 cells in bulk samples at all the considered time points.
