# Supplementary material for: Incorporation/Enrichment of 3D Bioprinted Constructs by Biomimetic Nanoparticles: Tuning Printability and Cell Behavior in Bone Models
Source: Nanomaterials (Basel). 2023 Jul 10;13(14):2040. doi: 10.3390/nano13142040 (PMC10386079; doi:10.3390/nano13142040)
Supplement: Supplementary file 1 [file nanomaterials-13-02040-s001.zip › nanomaterials-2464188-supplementary.pdf]

Evaluation of the metabolic activity of SAOS-2 cells embedded in bulk inks.

For all conditions, i.e. negligent of the nanoparticles and concentration (up to 2%), cells viability is maintained overtime and shows a positive trend until 14d of culture in all the samples with nHA and Sr-HA particles, with only Alg2SrHA showing a decrease at the last timepoint

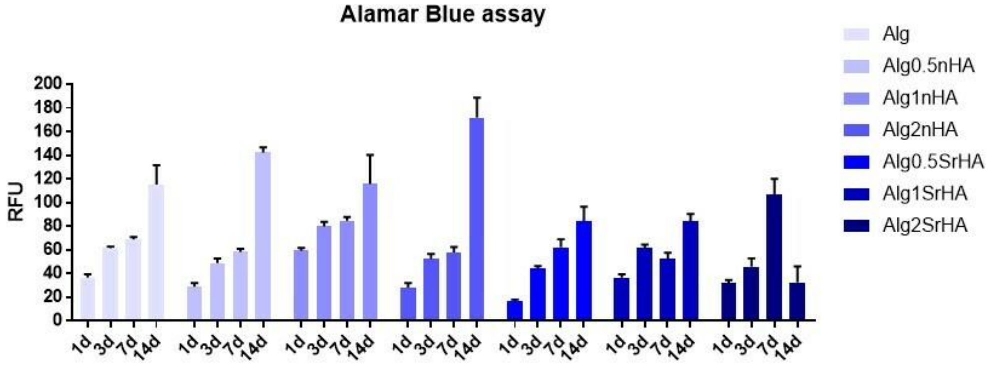

Figure S1. Metabolically active Saos-2 cells in bulk samples at all the considered timepoints (1d, 3d, 7d and 14d) (* p-value ≤ 0.05, ** p ≤ 0.01, *** p ≤ 0.001, **** p ≤ 0.0001). For 1d of culture: Alg vs Alg2nHA p=0.0001 ****; Alg vs Alg0.5SrHA p=0.0003 ***; Alg1nHA vsAlg0.5nHA p<0.0001 ****; Alg0.5nHA vs Alg0.5SrHA p=0.0022 **; Alg 1nHA vs Alg2nHA p<0.0001 ****; Alg1nHA vs Alg0.5SrHA p<0.0001 ****; Alg1nHA vs Alg1SrHA p=0.0001 ****; Alg1nHA vs Alg2SrHA p<0.0001 ****; Alg2nHA vs Alg0.5SrHA p=0.0067 **; Alg0.5SrHa vs Alg1SrHA p<0.0001 ****; Alg2SrHA vs Alg 0.5SrHA p=0.0004 ***. For 14d of culture: Alg vs Alg2nHA p=0.0428 *; Alg vs Alg0.5SrHA p=0.036 *; Alg vs Alg2SrHA p=0.0105 *; Alg1nHA vs Alg0.5nHA p=0.0377 *; Alg2nHA vs Alg0.5nHA p=0.0464*; Alg0.5nHA vs Alg0.5SrHA p=0.0007 ***; Alg0.5nHA vs Alg1SrHA p<0.0001 ****; Alg0.5nHA vs Alg2SrHA p<0.0001 ****; Alg1nha vs Alg2nHA p=0.0243*; Alg1nHA vs Alg0.5SrHA P=0.0153*; Alg1nHA vs Alg2SrHA p=0.0079 **; Alg2nHA vs Alg0.25SrHA p=0.0001 ****; Alg2nHA vs Alg1SrHA p<0.0001 ****; Alg2nHA vs Alg2SrHA p<0.0001 ****; Alg2SrHA vs Alf1SHA p=0.013 *.
